# Post-purchase behavioral intention in medical aesthetic: the role of image, perceived value, and satisfaction

**DOI:** 10.3389/fpubh.2024.1471496

**Published:** 2025-01-22

**Authors:** Chen-Chung Ma, Tzu-Chi Ou, Chun-Mei Tsai, Tai-Hsiang Chen

**Affiliations:** ^1^Department of Healthcare Administration, I-Shou University, Kaohsiung, Taiwan; ^2^Department of Medical Education, New Taipei Municipal Tucheng Hospital (Built and Operated by Chang Gung Medical Foundation), New Taipei, Taiwan; ^3^White Jade Medical Cosmetology Group, Kaohsiung, Taiwan; ^4^Department of Healthcare Administration, Linkou Chang Gung Memorial Hospital, Taoyuan, Taiwan

**Keywords:** medical aesthetic image, perceived value, satisfaction, post-purchase behavioral intention, medical beauty

## Abstract

**Introduction:**

The technicality and professionalism of medical aesthetics have become one of the most fashionable indicators in the world, and image is the key factor of aesthetic development. This study aims to explore the relationship between medical aesthetic image, perceived value, satisfaction and post-purchase behavioral intention.

**Methods:**

The subjects included customers who had consumed and received treatment in the three years, and the questionnaires were collected from returning clients of each unit in the medical aesthetic group from May 17, 2017 to May 31, 2017. We used the structural equation modeling (SEM) to analyze the data.

**Results:**

The results showed that medical aesthetic image was positively associated with perceived value, satisfaction and post-purchase behavioral intention; perceived value was positively associated with satisfaction and post-purchase behavioral intention; satisfaction was positively associated with post-purchase behavioral intention.

**Discussion:**

This study can provide medical aesthetic practitioners with an understanding of consumers' image and perceived value to improve satisfaction. The changes in consumers' perception of aesthetic medicine can be evaluated more objectively, and suggestions can be provided for medical institutions to strengthen their cultural connotation and external image, so as to establish a strong brand image.

## 1 Introduction

In response to the rapid growth of the medical aesthetic industry, this sector has become one of the most rapidly developing mature industries in the 21st century. Beyond its technical and professional nature, the medical aesthetics industry has also become one of the most trendy global indicators and is highly appreciated by consumers (1). Medical aesthetics has become one of the most promising trend industries, thus inducing many groups and doctors to enter the medical aesthetics market, resulting in fierce competition (2). The changing concept of plastic surgery has attracted more young people and is becoming one of the main sources of consumption in the medical aesthetic market (3). Nowadays, the new generation has its own concept of beauty and has a higher degree of acceptance. In addition to the international media, the visibility of communication technology is becoming more and more prevalent, people have given the meaning of rebirth and renewal to plastic surgery in the course of life. The establishment of a post-purchase behavioral model of the aesthetic medicine industry, the willingness of customers to repurchase and the maintenance of the willingness to recommend is good for both sides (4). For the enterprise, you can get a stable source of profit, but also reduce transaction costs and reduce uncertainty. Therefore, it is necessary to explore the customer's post-purchase behavior intention and find out the relevant factors affecting it.

With the improvement of people's living standards and the increasing concern for beauty, medical aesthetic institutions have emerged to meet the public demand for beauty. The development of the biotechnology and medical industry has boosted the demand for medical aesthetic products and services (5). Image is a key factor in the development of medical aesthetics. The medical aesthetics industry uses publicity, promotions and other marketing methods to increase its members and stimulate consumption. The perceived value perspective focuses on the feelings and emotions generated during the process of experiencing the quality and experience of the service when consumers participate in aesthetic activities, which leads to the behavior of consumers (6). Although medical aesthetic products and services have become quite common, there is a relative lack of research on consumer behavior. Therefore, this study investigates the relationship pattern of consumers' post-purchase behavioral intention after consumption in medical aesthetic institutions.

With the global pursuit of beauty and the improvement of quality of life, the medical beauty industry has gradually become a rapidly developing and competitive market in major countries. Consumers see medical beauty as a symbol of self-improvement and social status, a trend that reflects the Chinese market's strong desire for personalized beauty needs. In addition, with the improvement of living standards, the demand for cosmetic technology in the Chinese market is also growing, especially in minimally invasive surgery and skin management (7). At the same time, as an important market in the field of medical beauty, South Korea's government's strict supervision of the medical beauty industry has also prompted consumers to have higher requirements for the safety and quality of medical beauty services. The Korean market is particularly focused on advanced medical technology and equipment, and laser cosmetic and minimally invasive surgery have received extremely high attention and demand locally. These trends not only reflect Korean consumers' pursuit of high-quality medical services, but also highlight the important role of technological innovation in promoting market development (8). In the United States, the market of medical beauty is becoming increasingly competitive. As technology advances and consumers have higher expectations for service quality, more and more small clinics are relying on digital marketing and big data analytics to attract customers. Through precise market positioning and customer needs analysis, these clinics have successfully attracted a large number of consumers who value personalization and results, which has become one of their competitive advantages. The American medical beauty market provides many valuable inspirations in terms of technological innovation and the diversification of consumer demand levels (9). According to the development trend of the global medical beauty industry, we can foresee that the Taiwan market is also experiencing similar changes and challenges (10). With the growth of global consumer demand for medical beauty services, Taiwan's medical beauty institutions are also actively adjusting their strategies, not only strengthening the quality of service, but also introducing more advanced technology and equipment to cope with competitive pressure. The Taiwan market is characterized by the growing demand of young consumers, who have high expectations for personalization, aesthetics and quality of service (11). Therefore, medical beauty institutions in Taiwan should focus on improving customer experience and strengthening the establishment of brand image. Consumers in Taiwan have relatively high requirements for the quality and safety of beauty services, and with the rise of social media, consumer awareness and acceptance of medical beauty has increased significantly (10). This has made the medical beauty market in Taiwan increasingly competitive and prompted major medical beauty institutions to pay more attention to service quality and technological innovation to meet the increasingly diverse needs of consumers.

In the past research, product or service image was a relatively abstract concept, formed by the information received by customers and their previous consumption experiences (12). In tourism and leisure research, the concept of image was also frequently used to explain tourist destination choice and destination-related marketing, and some studies have confirmed that image is an important influence not only in the decision making process of potential consumers, but also on post-consumer behavior (13). In this study, the customers of medical aesthetic groups were selected as the target population. We proposed that operators of medical aesthetic institutions and future operators of related industries should understand consumers' imagination and perceived value of medical beauty and give practical suggestions. For example, what kind of specific experiential activities should be added, original service experience activities should be promoted, and existing facilities and resources should be utilized to shape the medical aesthetic industry's own style and attractiveness. Therefore, the purpose of this study was to explore the relationship between medical aesthetic image, perceived value, satisfaction, and post-purchase behavioral intention as practical suggestions for medical institutions to enhance brand awareness and establish a good brand image.

## 2 Literature review

### 2.1 Medical aesthetic image

Medical aesthetic image refers to the “attitudes, thoughts, and impressions that aesthetic medicine conveys.” Customers' perceptions of products or services influence consumer decisions (14, 15), and knowing and evaluating product and service image helps to further understand and predict consumer behavior (16). Medical aesthetic industry can provide both intangible services and tangible products, unlike tangible products that are generally easier to measure (17). How to create a positive, clear, enough to attract customers' image in the medical aesthetic industry is even more important. In general, the medical aesthetic image refers to customers' perception of the medical aesthetic group to provide the environment, medical staff skills, services, accessibility, product quality, information provision and corporate image.

### 2.2 Perceived value

Perceived value is a subjective evaluation of the balance between consumers' feeling, payment and acquisition, as well as an overall evaluation of product utility (18, 19). Dodds et al. (20) pointed out that perceived value is a trade-off relationship between perceived benefits and perceived value sacrifice when consumers produce their purchase intention. In other words, consumers do not blindly pursue the highest perceived quality when buying products, but obtain the highest perceived value in the transaction process that consumers are willing to pay (21). For the medical aesthetic industry, the evaluation of perceived value is not limited to the monetary transaction, but more important may be the subjective value of the overall service experience.

### 2.3 Satisfaction

Satisfaction is a major determinant of long-term customer behavior and an important indicator to measure future customer behavior (22). Therefore, satisfaction is the key factor leading to the change of experience attitude, which is the degree of enjoyment or disappointment caused by the expectation of the product (23). Scholars have different definitions of the concept of satisfaction, but most agree that the concept is complex and includes not only cognitive and emotional aspects, but also physiological and psychological dynamic factors (24). In this study, satisfaction was defined as the customer's assessment of the overall performance of the medical aesthetic group to their desired degree after using the products and services. The criteria for satisfaction evaluation include goods, treatment, system efficiency within the institution and service attitude and professional knowledge of medical staff.

### 2.4 Post-purchase behavioral intention

Behavioral intention refers to the possible tendency to act in the future, which can be used to predict people's behavior. Customers' satisfaction or dissatisfaction with the products they choose will affect their subsequent consumption behavior (25). Zeithaml et al. (26) believed that good service quality can enable customers to generate positive behavioral intention, such as good reputation, customer loyalty and publicity to others. Post-purchase behavioral intention can be used to predict whether consumers could become long-term customers and whether they could bring stable profits to enterprises (27). Generally speaking, the post-purchase behavioral intention refers to the possibility that consumers may influence their repurchase intention, payment intention and recommendation intention after the experience (28).

## 3 Conceptual model and hypotheses

**Figure 1:**
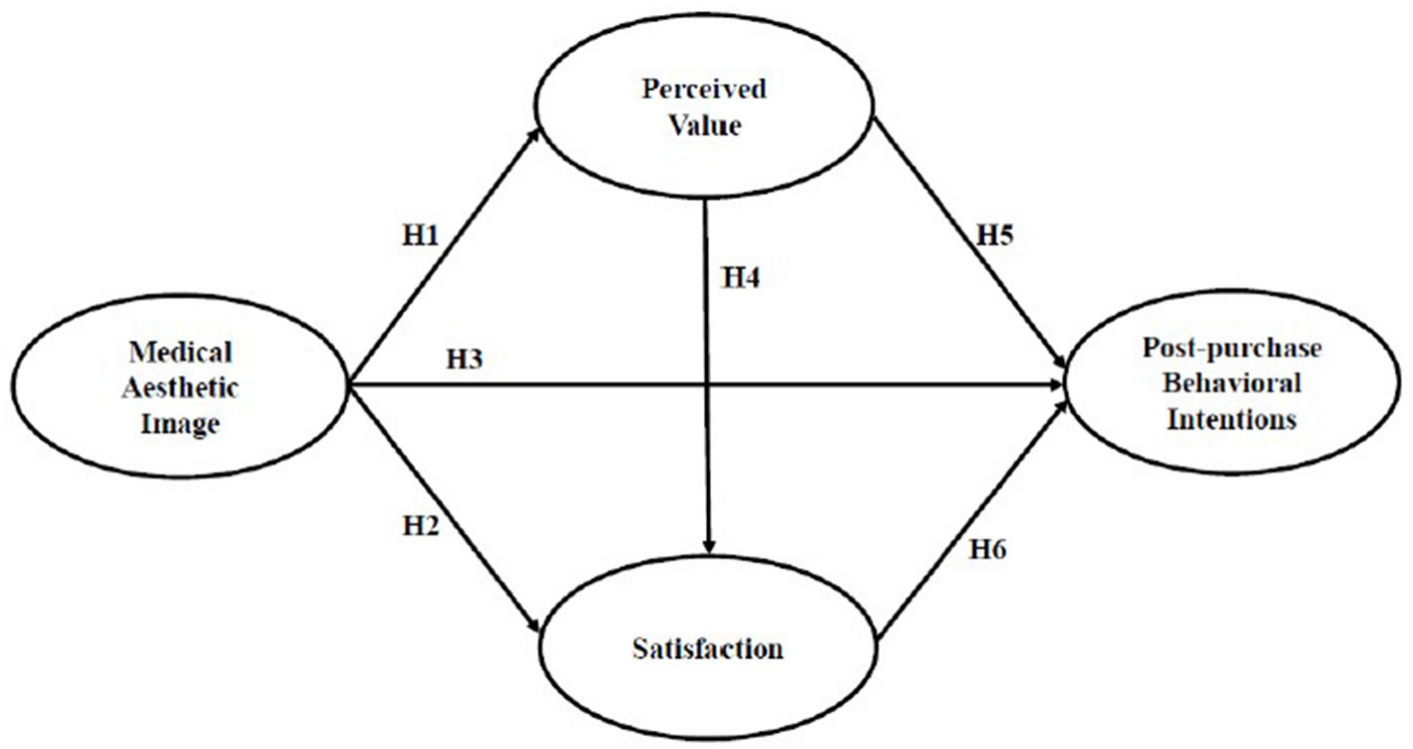
Research framework.

Previous studies have explored important concepts related to perceived value, customer satisfaction, and behavioral intention (26, 29–31). This study links the variables explored in previous studies and proposes a research framework that suggests that image affects consumers' own perceived value and satisfaction, and further influences their post-purchase behavioral intention. Aesthetic medicine is one of the booming industries that can help consumers achieve a beautiful appearance, and the value and profits of this industry are increasing dramatically every year, which makes the study of aesthetic medicine consumer behavior even more important (32). In addition, in the huge market scale of aesthetic medicine, the consumer behavior of customers is no longer passive acceptance, but has transformed into active need and participation in the various consumer activities of medical aesthetic institutions. They hope to get a humanized service environment and more professional consultation (33). In the context of this study, if consumers can have an image of medical aesthetic institutions through the environment, corporate image, and staff services. The more positive consumers feel about the image of the medical aesthetic group, the higher their perceived value and satisfaction will be. Therefore, a research framework was established as shown in Figure 1.

## 4 Medical aesthetic image and perceived value

With better image of the consumer environment, products or services can be shaped into high value perceptions first. Lien et al. (34) investigated the relationship between brand image and perceived value for consumers in Taiwan and found that consumers' image of brands had a positive relationship with perceived value, and that enhancing brand image would effectively increase the value of products/services. In a previous study on the relationship between product image and perceived value, the beauty industry was also used as an example, and it was found that corporate image has a correlation with perceived quality and customer value, which in turn affects customer loyalty (35). On the basis of these above discussions, we propose the following hypotheses:

H1: Medical aesthetic image is positively associated with perceived value.

## 5 Medical aesthetic image and satisfaction

Loudon and Della Bitta (36) pointed out that a good corporate image is likely to gain consumers' goodwill and trust; in other words, positive corporate image could increase customer satisfaction and loyalty. Font (37) suggested that image has a positive relationship with perceptual quality and satisfaction. Image shapes an individual's pre-consumption expectations. Quality and satisfaction are the result of comparing the actual experience with the original expectation, which also indicates that image and satisfaction may have a mutual influence on each other (38). A previous study investigated the effect of brand image on satisfaction and loyalty with consumers of aesthetic medicine and the results were positive and valid (39). On the basis of these above discussions, we propose the following hypotheses:

H2: Medical aesthetic image is positively with associated satisfaction.

## 6 Medical aesthetic image and post-purchase behavioral intention

A previous study suggested that the main reason affecting consumers' perceptions and satisfaction with quality is image, which is expressed through satisfaction levels and further influences consumers' related post-purchase behaviors (40). Therefore, rational consumers are more satisfied with their consumer experience through psychological and environmental expectations. Post-consumer image is a way of expressing the consumer experience and will influence consumer behavior. Court and Lupton (41) found that environmental image positively influenced consumers' willingness to return in the future. A previous study emphasized that a good image of a company can enhance customers' behavioral intention. The past study examined the impact of beauty industry image on consumers' repurchase behavior and found that the impression of functional value brought by beauty products had a positive relationship with post-purchase behavioral intention (42). On the basis of these above discussions, we propose the following hypotheses:

H3: Medical aesthetic image is positively associated with post-purchase behavioral intention.

## 7 Perceived value and satisfaction

Anderson and Sullivan (43) defined that the level of perceived value directly affects customer satisfaction with the service provider; in other words, satisfaction is the result of how customers feel after receiving the perceived value (44). Both Bolton and Drew (45) and Woodruff (46) pointed out that perceived value must be assessed before satisfaction. If this is not done, customer-oriented measurement may be lacking. This may result in the product or service being strategized and arranged differently from the actual situation. Chen and Hu (47) found that perceived value had a statistically significant positive correlation with loyalty, and perceived value also had a positive and significant effect on satisfaction in the Australian coffee industry. On the basis of these above discussions, we propose the following hypotheses:

H4: Perceived value is positively associated with satisfaction.

## 8 Perceived value and post-purchase behavioral intention

Grewal et al. (19) suggested that perceived value affects consumers' internal reference prices. In addition, transactional value influences consumers' purchase intentions and search intentions by obtaining perceived value. The empirical results of McCarthy and Perreault (48) and Schiffman and Kanuk (49) showed that consumers' perceived value affects the willingness to purchase a product. It can be inferred that the degree of consumer experience affects the outcome of perceived value, which in turn affects customer behavioral intention. On the basis of these above discussions, we propose the following hypotheses:

H5: Perceived value is positively associated with post-purchase behavioral intention.

## 9 Satisfaction and post-purchase behavioral intention

Yi and La (50) proposed that repurchase behavior intention is positively influenced by customer satisfaction. Kotler and Keller (23) suggested that customers with high levels of pleasure or satisfaction will have an emotional attachment to the brand, which further influences behavioral intention. Therefore, when customers have high repurchase intentions and satisfaction levels, they will usually use aesthetic services again and are more likely to be loyal customers. By maintaining customer loyalty, we can increase this group's willingness to repurchase, which will have a positive effect on behavioral intention (51). On the basis of these above discussions, we propose the following hypotheses:

H6: Satisfaction is positively associated with post-purchase behavioral intention.

## 10 Methodology

Based on the concept of consumer behavior, this study investigated the relationship between medical aesthetic image, perceived value, satisfaction, and post-purchase behavioral intention. In 2014–2017, the global economy was still in the post-financial crisis recovery stage, and the market turbulence had an intricate and complex impact on consumer behavior and industry development, especially the medical aesthetic industry (52, 53). The development of the medical aesthetics market was reshaped against the backdrop of an economic environment that resembled an intricate economic canvas in which every stroke could profoundly affect consumer choices and corporate strategies. On the technological front, social media and digitalization have created a colorful era that has not only changed consumer needs, but also forced companies to reconstruct their marketing and service models in the face of competition. With the penetration of artificial intelligence, the medical aesthetic industry is beginning to enter a new phase, pregnant with unlimited opportunities. The cross-sectional study design was adopted. The target demographic includes medical beauty consumers in southern Taiwan, who first visited from 2014 to 2016 and again in May 2017. Questionnaires were distributed to clients who received treatment during this period. The contents of the survey include four parts: “medical aesthetic image,” “perceived value,” “customer satisfaction,” and “post-purchase behavior intention.” Paper questionnaires were used for purposeful sampling, and questionnaires were conducted among returning customers from May 17 to May 31, 2017. A total of 350 questionnaires were sent out and 312 were returned. After excluding five invalid questionnaires, 307 valid questionnaires were retained, with an effective recovery rate of 88%.

According to the purpose and structure of the study, we designed the research questionnaire with reference to past literature. After the first draft of the questionnaire was completed, five experts in the field of medical aesthetics and healthcare were invited to review, including a regional hospital director, a medical center director, a medical aesthetic facility director, a medical aesthetic group marketing manager, and an associate professor in the department of healthcare administration. The questionnaire was reviewed and revised question by question for appropriate content validity. In terms of the medical aesthetic image, four items are adaptations from Prayag and Ryan (54) and were modified to fit the background of medical aesthetic group. To measure the perceived value of a medical aesthetic experience, this study used three items on the basis of the research of Petrick (55). Measurement of satisfaction employed three items on the basis of studies by Oliver (22) and Hellier et al. (56). Finally, the instrument for behavioral intention was measured using three items adapted from Zeithaml et al. (26). Excluding demographic questions, all items were based on a 7-point Likert-type scale (eg, 1 = strongly disagree, and 7 = strongly agree). The collected data were analyzed using SPSS 22 statistical software for basic descriptive statistics, and structural equation modeling (SEM) was used to test the research hypotheses with AMOS 23 software. To assess the reliability of the measurement model, factor loadings, composite reliability (CR), average variance extracted (AVE), and Cronbach's α were calculated. The threshold values of 0.7 for factor loadings and 0.7 for CR and Cronbach's α, and 0.5 for AVE, were used as recommended by Hair et al. (57). Convergent validity was assessed by evaluating the AVE, ensuring it was >0.7, while discriminant validity was confirmed by checking that the square root of the AVE for each construct was higher than the correlation between that construct and others, as suggested by Fornell and Larcker (58). The structural model was tested using SEM to assess the hypothesized relationships between constructs. Model fit was evaluated using fit indices such as χ^2^/df, GFI, AGFI, RMSEA, CFI, and NFI, with appropriate thresholds defined in the literature [e.g., Singh (59)].

The institutional review board (IRB) of the Kaohsiung Armed Forces General Hospital approved this study (KAFGH 106-015). This study does not involve live animals and individual human participants. The requirement for informed consent from the study subjects was waived by the IRB of the Kaohsiung Armed Forces General Hospital due to the reason that the data analyzed in this study are de-identification, anonymized, and aggregated within the hospital. No personally identifiable information, such as an individual's location, contacts, or movement, was made available at any point. This study confirms that all methods were performed in accordance with the relevant guidelines and regulations. The datasets analyzed during the current study are not publicly available due privacy of the patients but are available from the corresponding author on reasonable request.

## 11 Data analysis and results

### 11.1 Demographic information

The collected data were analyzed by SPSS 22 statistical software for basic data, and the research hypotheses were validated by structural equation model and analyzed by AMOS 23 software. As shown in Table 1, regarding the demographic information of the respondents, the majority of the respondents were female (86.6%), and the majority of the respondents were aged 21–40 (71.3%). 58.3% of the respondents were married, and 60.3% had no children. The majority of respondents (64.8%) had a university degree or higher, and about half of them worked in the private enterprise (54.4%).

**Table 1:** Demographic information of respondents.

**Demographic information**	**Categories**	**Cases**	**Percentage**
Sex	Female	266	86.6
	Male	41	13.4
Age	18–20	21	6.8
	21–30	97	31.6
	31–40	122	39.7
	41–50	55	17.9
	51–60	8	2.6
	≧61	4	1.3
Marital status	Married	179	58.3
	Unmarried	118	38.4
	Other (including divorced, widowed, separated, etc.)	10	3.3
Children or not	No	185	60.3
	Yes	122	39.7
Highest education	Master or above	48	15.6
	University	151	49.2
	Junior college	37	12.1
	High school	67	21.8
	Junior high school or below	4	1.3
Occupation	Public sector	42	13.7
	Private enterprise	167	54.4
	Freelance	59	19.2
	Self-employment	25	8.1
	Home Management	6	2.0
	Student	8	2.6

### 11.2 Measurement model

As suggested by Hair et al. (57), this study utilized individual questions factor loading, composite reliability (CR), average variance extraction (AVE), and Cronbach's α to assess reliability. We compiled the results in Table 2. First, according to Ibrahim et al. (60), the factor loading must be higher than the standard 0.7. The results of the analysis showed that the factor loading of each question for medical aesthetic image, perceived value, satisfaction and post-purchase behavioral intention were higher than the standard 0.7. In addition, according to Hair et al. (57), the CR and Cronbach's α should be higher than the standard 0.7 and the AVE should be higher than the standard 0.5. The results showed that the CR was higher than 0.9 and the AVE was higher than 0.7, both of which were above the standard, indicating that the constructs and measurement variables of this study had sufficient reliability.

**Table 2:** Reliability and validity analysis.

**Constructs/items**	**Factor loading**	**AVE**	**CR**	**Cronbach α**
**Medical aesthetic image**				
The medical aesthetic group provides good service	0.747	0.719	0.910	0.919
The medical aesthetic group has good accessibility from home	0.890			
The medical aesthetic group has good reputation	0.840			
The medical aesthetic group has professional feature	0.905			
**Perceived value**				
Fees were fairly priced at this medical aesthetic group	0.861	0.791	0.938	0.935
Quality of service at this medical aesthetic group has a good reputation	0.912			
Overall quality of the service was valuable	0.852			
**Satisfaction**				
All things considered, I feel good about my decision to consume in this medical aesthetic group	0.804	0.797	0.940	0.938
Overall, I am satisfied with everything here	0.897			
Considering all my experience with this consumption, my choice to this medical aesthetic group was a wise one	0.955			
**Post-purchase behavioral intention**				
I would like to return to this medical aesthetic group in the future	0.707	0.792	0.927	0.922
I would recommend this medical aesthetic group to my friends or other acquaintances	0.953			
I want to tell other people positive things about this medical aesthetic group	0.888			

Furthermore, the validity was assessed by convergent validity and discriminant validity, and the results were shown in Table 2. The AVE was used to interpret and measure the extent of potential variables, as suggested by Fornell and Larcker (58). The analysis of this study showed that the AVE was higher than 0.7 for all components. The criterion higher than 0.5 indicates adequate convergent validity. The discriminant validity was attained if the square root of AVE for each construct is higher than the correlations between the construct and the other constructs (58). As shown in Table 3, the square root of AVE for each construct was between 0.80–0.90, which was higher than the correlation coefficient between the constructs. Then it could be inferred that the theoretical model had sufficient discriminant validity. After the above, the study had sufficient reliability and validity to proceed to the next step of structural model analysis.

**Table 3:** Discriminant validity analysis.

**Variables**	**1**	**2**	**3**	**4**
1. Medical aesthetic image	0.848			
2. Perceived value	0.727^**^	0.889		
3. Satisfaction	0.717^**^	0.813^**^	0.893	
4. Post-purchase behavioral intention	0.674^**^	0.771^**^	0.736^**^	0.890

^**^p < 0.01.

### 11.3 Structural model

The structural model evaluated the path coefficients between constructs based on directionality and significant correlation. The structural equation model was constructed to test the hypothesis, and the model fitness was particularly important. In this study, we referred to the model fitness index proposed by Singh (59). Estimated fit indices seem to show a good model fit, in particular: χ^2^/df = 2.311 (*p* < 0.01); GFI = 0.915; AGFI = 0.883; RMSEA = 0.065; CFI = 0.975; NFI = 0.969.

**Figure 2:**
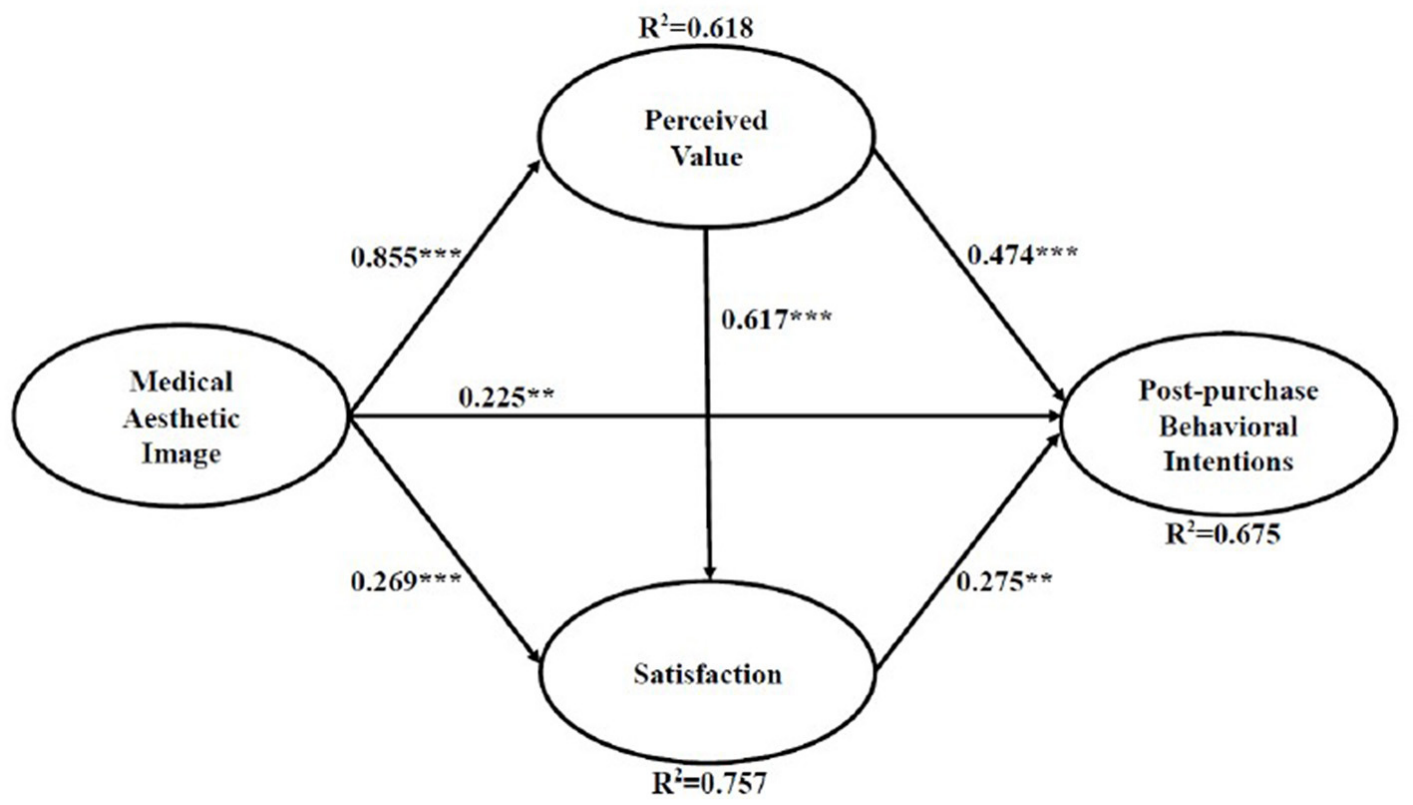
Path coefficient analysis. **p < 0.01, ***p < 0.001.

The final structural model with the estimated standardized path coefficients and path significance among the constructs was presented in Figure 2 and Table 4. As hypothesized, all of the proposed hypotheses were supported. Perceived value was significantly influenced by medical aesthetic image (β = 0.855, *p* < 0.001). Satisfaction was significantly influenced by medical aesthetic image (β = 0.269, *p* < 0.001) and perceived value (β = 0.617, *p* < 0.001), providing support for H2 and H4. Post-purchase behavioral intention was significantly influenced by medical aesthetic image (β = 0.225, *p* < 0.01), perceived value (β = 0.474, *p* < 0.001) and satisfaction (β = 0.275, *p* < 0.01), providing support for H3, H5, and H6. Overall, the model explained about 61.8%, 75.7%, and 67.5% of the determined variance in the perceived value, satisfaction and post-purchase behavioral intention, respectively.

**Table 4:** Research hypothesis validation.

**Hypothesis**	**Path coefficient**	***t*-value**	**Results**
H1 Medical aesthetic image → perceived value	0.855^***^	15.07	Supported
H2 Medical aesthetic image → satisfaction	0.269^***^	4.24	Supported
H3 Medical aesthetic image → post-purchase behavioral intention	0.225^**^	2.73	Supported
H4 Perceived value → satisfaction	0.617^***^	9.97	Supported
H5 Perceived value → post-purchase behavioral intention	0.474^***^	4.87	Supported
H6 Satisfaction → post-purchase behavioral intention	0.275^**^	2.85	Supported

^**^p < 0.01.

^***^p < 0.001.

## 12 Discussion

Based on the statistical analysis of the questionnaires, it was found that medical aesthetic image had a significant positive effect on perceived value, which was consistent with the findings of Lien et al. (34). They investigated the relationship between brand image and perceived value in the hotel industry in Taiwan, and found that consumers' image of corporate brands affects their perception of the value and quality of products and services. In this study, it was inferred that consumers would generate business images of medical aesthetic institutions from the media or recommendations from friends and relatives. The image would have an impact on the overall perceived value judgment. Therefore, good image not only affected consumers' awareness of the medical aesthetic industry, but also affected their good feelings and evaluation of medical aesthetic institutions. In addition, this result was also consistent with the findings of Marcela Betancur et al. (39), which found that consumers' image and perceived value of medical aesthetic institutions had a significant positive correlation. The image and perception of aesthetic medicine had a direct effect on the overall perceptual feeling. If the medical aesthetic industry can create a good image, consumers will be able to judge a good value feeling. The image of aesthetic medicine will naturally improve, and can also improve the perceived value, which was the key point of the medical aesthetic industry should not be neglected in business. The results of this study further confirmed that consumers evaluate the quality of products and services in the process of consumption. If the quality exceeds their expectations, they will further compare it with the sacrifice they have paid to generate a feeling of value. Therefore, for providers of medical aesthetic products and services, to provide high-quality products or reduce the cost of consumer perceptions, can effectively enhance the perceived value of consumers.

As for the significant positive effect of medical aesthetic image on satisfaction, the structural model suggests that medical aesthetic image would be an important factor in predicting consumer satisfaction with medical aesthetic products and services. This result was consistent with previous literature that examined the association between hospital image and satisfaction (61). The findings of this study also support the viewpoint of Anderson et al. (62) on customer satisfaction. It is proposed that satisfaction can be divided into transaction-specific and cumulative types. The former refers to the customer's prediction of the service provided by a particular service provider before assessing the satisfaction level. In cumulative satisfaction, the customer's expectation is derived from the experience of the previous vendor's product and then predicts the service in the future. In these two types of satisfaction perspectives, customer satisfaction with a particular provider is derived from information, experience, and predictions of the customer's previous service performance. Similar to the concept of medical aesthetic image mentioned in this study, it shows that the more positive the comprehensive perceptual image of the environment, staff service, product quality, and corporate image provided by medical aesthetic institutions, the more customer satisfaction will be significantly increased.

In addition, based on the findings, this study confirmed a significant positive effect of medical aesthetic image on post-purchase behavioral intention, which was similar with previous study examining the relationship between consumer image and post-purchase behavioral intention for medical aesthetic cosmetic stores (63). The study investigated the effect of corporate image of medical aesthetic stores on post-purchase behavioral intention of private brands and found that the better the consumer's pre-consumer image, the more positive the consumer's post-purchase behavioral intention. Before consumption, the information that consumers receive during the construction of the image of medical aesthetics becomes a kind of comparative criterion and is constantly compared with the experience during the consumption process. This image continues to exist until the end of the consumption or experience, and is then modified from this experience. Therefore, consumers' continuous aesthetic imagination will affect their post-purchase behavior, which in turn will influence whether they will buy again or recommend to others in the future. The past study found that the consumer experience belongs to the stage of service interaction in the consumer purchase process, which usually occurs before the purchase of the product behavior. It is further deduced that after receiving the service or experience of medical aesthetic products, consumers will derive feelings of satisfaction and influence the occurrence of subsequent post-purchase behaviors (64).

The present study also confirmed a positive and statistically significant association between perceived value and satisfaction, which was consistent with the results of a previous study that examined the relationship between perceived value and customer satisfaction (65). The empirical results of this study found that consumer perceived value has a direct positive effect on satisfaction. When consumers purchase aesthetic treatments, a good perceived value increases post-purchase satisfaction relatively. Perceived value represents how they feel when the cost is compared to the benefit they receive (66). Therefore, in the marketing of medical aesthetic products, the enhancement of consumers' perceived value is an important key to increase their satisfaction. Chen and Hu (47) found that there is a statistically significant correlation between perceived value and customer satisfaction. Increased satisfaction is when customers perceive that the ratio of gains to losses is better than originally expected.

This study demonstrates that perceived value has a significant positive effect on post-purchase behavioral intention. This may be due to the fact that when consumers feel that the experience at the facility is worthwhile, it motivates them to purchase the product, and they may share the experience with their friends at the facility and invite them to the next visit. Therefore, perceived value affects loyalty and post-purchase behavior, which is the same as the empirical findings of Wu et al. (67). They surveyed 887 online shoppers of medical aesthetic products and found that there was a significant positive relationship between consumers' perceived value and post-purchase behavioral intention. The higher the perceived value of a customer of a medical aesthetic organization, the more the customer agrees with the organization. In this way, it is possible to predict behavioral intention such as repurchase, sharing intentions, and recommending others.

Based on the analysis, this study found a positive and statistically significant association between satisfaction and post-purchase behavioral intention, similar to the results of a previous study (68). They confirmed the correlation between satisfaction and post-purchase behavioral intention among 513 Macau consumers. Secondly, an effect on recommendation intention was also found. In this study, satisfaction is the psychological state of consumers after their needs are met. If consumers feel satisfied, it means that there are certain treatments or things that can satisfy their needs in medical aesthetic institutions. In the future, if there is the same demand, or others have similar needs, there will be repurchase or recommend others to come to spend. In other words, this also belongs to the performance of loyalty, so it is clear that in the marketing of aesthetic medicine products and services, the goal is to strive to improve consumer satisfaction. This is an effective way to increase their post-purchase positive behavioral intention.

## 13 Conclusions

Although this study emphasizes the rigorous process of research design, methodology, and data analysis, there are still some research limitations. In this study, the sampling method was purposive sampling, which was conducted in the lobby of a medical aesthetic group in southern Taiwan, where the customers were explained and asked if they were willing to help fill in the questions. The choice of location may be an obstacle, and other more representative samples of customers in the medical aesthetic group may be missed. In addition, purposive sampling may lead to a tendency for researchers to select respondents, resulting in sampling bias in the study model. On the other hand, this study was limited to a medical aesthetic group in southern Taiwan, so the results of the study cannot be fully extrapolated to all the medical aesthetic institutions in all regions of Taiwan. The fact that some consumers in this study have received their previous treatment for nearly 3 years may result in response errors due to the longer period of time.

The results of this study are limited by the cross-sectional study design and the absence of pre and post-test data for the same study sample. Therefore, it is not possible to explain the changes in consumers' image, perceived value, satisfaction, and post-purchase behavioral intention toward the medical aesthetic group before and after the consumer experience or purchase of the product. Alternatively, a longitudinal study may be conducted to examine the effects of continuity and later behavioral development. In addition, we used a quantitative research method to conduct the study. It is suggested that future studies can be conducted both qualitatively and quantitatively, with a deeper understanding and exploration from the perspective of aesthetic medicine practitioners and consumers. Future research can also expand the scope of the study to compare the differences between more equivalent medical aesthetic institutions, in order to make the research results more representative.

Medical aesthetic image may create a similar preconceived notion among consumers, which can have a positive impact on the evaluation of product value and post-purchase behavioral intention (13). If medical aesthetic practitioners want to build a good image, they must promote various sources of information that can enhance the image, such as TV commercials, program reports, joint promotion with other medical aesthetic related industries, and even teaching and research work. Of course, the most important thing is to maintain consistent product and service standards, so that customers who have received treatment can promote the medical aesthetic organization. However, it is not advisable to advertise, embellish, or even exaggerate the content of medical products, as this will not only be unattractive to consumers, but will also have the opposite effect. The medical aesthetic industry has long been dedicated to technical expertise, but this business model is no longer compatible with the current competitive market environment. In addition to technical expertise, good quality of care requires more comprehensive health care services (69), so providers can allow customers to participate in the evaluation of the entire medical process after completing the treatment and encourage consumers to propose solutions to their dissatisfaction. In this way, the blind spots in the treatment or service can be remedied, and the satisfaction of consumers can be improved. Perceived value can be expanded to include education and training. For example, the medical aesthetic industry can provide training certificates and arrange teaching courses on medical aesthetic techniques, products, and aesthetics. It can increase consumer satisfaction by enhancing their perceived value, as well as strengthening their repurchase and word-of-mouth promotion effects to build a truly sustainable and differentiated competitive advantage.

The conclusions of this study correspond to the actual business situation of the medical aesthetic group in this study. In order to build a good image of the medical aesthetic industry, they will advertise through online operations and print publicity, such as hiring models to advertise on their behalf, and often hold public service seminars and orphanage visits. Most of the medical aesthetics industry does not use the low profit promotion to attract consumers, but proposes high price and high quality marketing strategies, such as hiring more professional and comprehensive doctors to perform cosmetic surgery. Alternatively, the professionalism and service attitude of the medical staff can be enhanced so that consumers can understand the value of the products and services provided by medical aesthetic institutions. The medical aesthetics industry not only pays attention to consumers' response to products at all times, but also continues to improve product quality and value. We hope that our high standard of staff service will make our customers satisfied before, during and after their consumption. This will enhance consumers' sense of security and trust in the medical aesthetics group and lead to more subsequent consumption behavior. In summary, this study confirms that if medical aesthetic operators can establish a positive image, it will effectively increase customers' perceived value and satisfaction of medical aesthetic products and services, and further increase post-purchase behavioral intention.

## Data Availability

The original contributions presented in the study are included in the article/Supplementary material, further inquiries can be directed to the corresponding author.

## References

[1] Wright DWM Zascerinska S. Becoming immortal: future wellness and medical tourism markets. J Tour Futures. (2023) 9:168–95. 10.1108/JTF-05-2021-0119

[2] Lin YY Lean HH Lan HY Lee TR. Will my customers come back? A study of beauty salons in Taiwan. J Distrib Sci. (2018) 16:73–85. 10.15722/jds.16.1.201801.73

[3] Edmonds A. The poor have the right to be beautiful: cosmetic surgery in neoliberal Brazil. J R Anthropol Inst. (2007) 13:363–81. 10.1111/j.1467-9655.2007.00427.x

[4] Liu Z. “Zhuge Kongming becomes reborn as a clownish partygoer!”: linguistic carnivalization, critical metapragmatics of danmu, and mediatized neoliberal (inter) subjectivity. Discourse Context Media. (2024) 61:100815. 10.1016/j.dcm.2024.100815

[5] Kuipers G. The expanding beauty regime: or, why it has become so important to look good. Crit Stud Fash Beauty. (2022) 13:207–28. 10.1386/csfb_00046_1

[6] Abid MF Shamim A Khan Z Khan I. Value creation or value destruction: conceptualizing the experiential nature of value-in-use. J Consum Behav. (2022) 21:583–601. 10.1002/cb.2033

[7] Li EPH Min HJ Lee S. Soft power and nation rebranding: the transformation of Korean national identity through cosmetic surgery tourism. Int Mark Rev. (2021) 38:141–62. 10.1108/IMR-01-2019-0053

[8] Gaeb AK Hussein AA Ismael AS Khader MD Khalaf MA. Advancements in medical devices for skin beautification. Eur J Modern Med Pract. (2024) 4:108–21. 10.5281/zenodo.13740350

[9] Guiry M Vequist DG. Traveling abroad for medical care: US medical tourists' expectations and perceptions of service quality. Health Mark Q. (2011) 28:253–69. 10.1080/07359683.2011.595644 21815742

[10] Nguyen THN Yeh QJ Huang CY. Understanding consumer' switching intention toward traceable agricultural products: push-pull-mooring perspective. Int J Consum Stud. (2022) 46:870–88. 10.1111/ijcs.12733

[11] Liao SH Widowati R Cheng CJ. Investigating Taiwan Instagram users' behaviors for social media and social commerce development. Entertain Comput. (2022) 40:100461. 10.1016/j.entcom.2021.100461

[12] Bigné JE Sánchez MI Sánchez J. Tourism image, evaluation variables and after purchase behaviour: inter-relationship. Tour Manag. (2001) 22:607–16. 10.1016/S0261-5177(01)00035-8

[13] Shen SL Lee MC Lin CY. The influence of brand image and perceived value of Cross-Border E-commerce businesses on consumer purchase intention with Thailand's Baggo serving as the study object. Int J Uncertain Innov Res. (2020) 2:57–71.

[14] Loureiro SMC Stylos N Miranda FJ. Exploring how mindfulness may enhance perceived value of travel experience. Serv Ind J. (2020) 40:800–24. 10.1080/02642069.2019.1600672

[15] Liu P Li M Dai D Guo L. The effects of social commerce environmental characteristics on customers' purchase intentions: the chain mediating effect of customer-to-customer interaction and customer-perceived value. Electron Commer Res Appl. (2021) 48:101073. 10.1016/j.elerap.2021.101073

[16] Alhemoud AM Armstrong EG. Image of tourism attractions in Kuwait. J Travel Res. (1996) 34:76–80. 10.1177/004728759603400413

[17] Haverila MJ Haverila K McLaughlin C Tran H. The impact of tangible and intangible rewards on online loyalty program, brand engagement, and attitudinal loyalty. J Mark Anal. (2022) 10:64–81. 10.1057/s41270-021-00150-7

[18] Zeithaml VA. Consumer perceptions of price, quality, and value: a means-end model and synthesis of evidence. J Mark. (1988) 52:2–22. 10.1177/002224298805200302

[19] Grewal D Monroe KB Krishnan R. The effects of price-comparison advertising on buyers' perceptions of acquisition value, transaction value, and behavioral intentions. J Mark. (1998) 62:46–59. 10.1177/002224299806200204

[20] Dodds WB Monroe KB Grewal D. Effects of price, brand, and store information on buyers' product evaluations. J Mark Res. (1991) 28:307–19. 10.1177/002224379102800305

[21] Maroufkhani P Asadi S Ghobakhloo M Jannesari MT Ismail WKW. How do interactive voice assistants build brands' loyalty? Technological Forecasting and Social Change (2022) 183:121870. 10.1016/j.techfore.2022.121870

[22] Oliver RL. A cognitive model of the antecedents and consequences of satisfaction decisions. J Mark Res. (1980) 17:460–9. 10.1177/00222437800170040523326940

[23] Kotler P Keller KL. Building customer satisfaction, value, and retention. In: Kotler P, editor. Marketing Management. (2006).

[24] Suhartanto D. Tourist satisfaction with souvenir shopping: evidence from Indonesian domestic tourists. Curr Issues Tourism. (2018) 21:663–79. 10.1080/13683500.2016.1265487

[25] Doney PM Cannon JP. An examination of the nature of trust in buyer–seller relationships. J Mark. (1997) 61:35–51. 10.1177/002224299706100203

[26] Zeithaml VA Berry LL Parasuraman A. The behavioral consequences of service quality. J Mark. (1996) 60:31–46. 10.1177/002224299606000203

[27] Chen CF Chen WY. A study on the relationship between image, perceived value, satisfaction and post-purchase behavioral intention of study tour for university students. J Outdoor Recreation Study. (2005) 18:23–46.

[28] Ma KX Mather DW Ott DL Fang E Bremer P Mirosa M. Fresh food online shopping repurchase intention: the role of post-purchase customer experience and corporate image. Int J Retail Distrib Manag. (2022) 50:206–28. 10.1108/IJRDM-04-2021-0184

[29] Cronin JJ Jr Brady MK Hult GTM. Assessing the effects of quality, value, and customer satisfaction on consumer behavioral intentions in service environments. J Retail. (2000) 76:193–218. 10.1016/S0022-4359(00)00028-2

[30] McDougall GHG Levesque T. Customer satisfaction with services: putting perceived value into the equation. J Serv Mark. (2000) 14:392–410. 10.1108/08876040010340937

[31] Petrick JF Backman SJ. An examination of the construct of perceived value for the prediction of golf travelers' intentions to revisit. J Travel Res. (2002) 41:38–45. 10.1177/0047287502041001005

[32] Craddock N Spotswood F Rumsey N Diedrichs PC. “We should educate the public that cosmetic procedures are as safe as normal medicine”: understanding corporate social responsibility from the perspective of the cosmetic procedures industry. Body Image. (2022) 43:75–86. 10.1016/j.bodyim.2022.08.011 36063763

[33] Iliffe S Manthorpe J. Medical consumerism and the modern patient: successful ageing, self-management and the ‘fantastic prosumer'. J R Soc Med. (2020) 113:339–45. 10.1177/0141076820911574 32910877 PMC7488811

[34] Lien CH Wen MJ Huang LC Wu KL. Online hotel booking: the effects of brand image, price, trust and value on purchase intentions. Asia Pac Manag Rev. (2015) 20:210–8. 10.1016/j.apmrv.2015.03.005

[35] Leung PPL Wu CH Ip WH Ho GTS. Enhancing online-to-offline specific customer loyalty in beauty industry. Enterp Inf Syst. (2019) 13:352–75. 10.1080/17517575.2018.1527042

[36] Loudon DL Della Bitta AJ. Consumer Behavior: Concepts and Applications. New York, NY: McGraw-Hill Companies (1984).

[37] Font X. Managing the tourist destination's image. J Vacation Mark. (1997) 3:123–31. 10.1177/135676679700300203

[38] Fu X Liu S Fang B Luo XR Cai S. How do expectations shape consumer satisfaction? An empirical study on knowledge products. J Electron Commerce Res. (2020) 21:1–20.

[39] Marcela Betancur D Montoya Castañeda K Tavera-Mesías JF. Correlational study of the factors that influence in the recommendation and loyalty of patients of aesthetic medicine Medellín Colombia, 2014. Cuad Adm. (2017) 33:3–17. 10.25100/cdea.v33i58.4527

[40] Kotler P. Marketing for Hospitality and Tourism, Global Edition. 7th Edn. London: Pearson Education (2016).

[41] Court B Lupton RA. Customer portfolio development: modeling destination adopters, inactives, and rejecters. J Travel Res. (1997) 36:35–43. 10.1177/004728759703600106

[42] Ahmad SNB Omar A. Influence of perceived value and personal values on consumers repurchase intention of natural beauty product. Int J Supply Chain Manag. (2018) 7:116–25.

[43] Anderson EW Sullivan MW. The antecedents and consequences of customer satisfaction for firms. Mark Sci. (1993) 12:125–43. 10.1287/mksc.12.2.12519642375

[44] Hallowell R. The relationships of customer satisfaction, customer loyalty, and profitability: an empirical study. Int J Serv Ind Manag. (1996) 7:27–42. 10.1108/09564239610129931

[45] Bolton RN Drew JH. A multistage model of customers' assessments of service quality and value. J Consum Res. (1991) 17:375. 10.1086/208564

[46] Woodruff RB. Customer value: the next source for competitive advantage. J Acad Mark Sci. (1997) 25:139–53. 10.1007/BF02894350

[47] Chen PT Hu HH. The effect of relational benefits on perceived value in relation to customer loyalty: an empirical study in the Australian coffee outlets industry. Int J Hosp Manag. (2010) 29:405–12. 10.1016/j.ijhm.2009.09.006

[48] McCarthy EJ Perreault WD. Basic Marketing: A Managerial Approach. Homewood, IL: Richard D Irwin (1984).

[49] Schiffman LG Kanuk LL. Consumer Behavior, 7th Edn. Hoboken, NJ: Prentice Hall (2000), p. 15–36.

[50] Yi Y La S. What influences the relationship between customer satisfaction and repurchase intention? Investigating the effects of adjusted expectations and customer loyalt. Psychol Mark. (2004) 21:351–73. 10.1002/mar.20009

[51] Molinillo S Aguilar-Illescas R Anaya-Sánchez R Liébana-Cabanillas F. Social commerce website design, perceived value and loyalty behavior intentions: the moderating roles of gender, age and frequency of use. J Retail Consum Serv. (2021) 63:102404. 10.1016/j.jretconser.2020.102404

[52] Pagán R Horsfall D. Medical tourism trends in the United Kingdom 2000-2016: global economic crisis, migration and UK expats under consideration. J Tour Anal. (2020) 27:20–40. 10.1108/JTA-06-2019-0025

[53] Hopkin J Blyth M. The global economics of European populism: growth regimes and party system change in Europe (The government and opposition/Leonard Schapiro lecture 2017). Gov Oppos. (2019) 54:193–225. 10.1017/gov.2018.43

[54] Prayag G Ryan C. Antecedents of tourists' loyalty to Mauritius: the role and influence of destination image, place attachment, personal involvement, and satisfaction. J Travel Res. (2012) 51:342–56. 10.1177/0047287511410321

[55] Petrick JF. Development of a multi-dimensional scale for measuring the perceived value of a service. J Leis Res. (2002) 34:119–34. 10.1080/00222216.2002.1194996524499259

[56] Hellier PK Geursen GM Carr RA Rickard JA. Customer repurchase intention: a general structural equation model. Eur J Mark. (2003) 37:1762–800. 10.1108/03090560310495456

[57] Hair JF Ringle CM Sarstedt M. Editorial - partial least squares structural equation modeling: Rigorous applications, better results and higher acceptance. (2013). Available at: https://papers.ssrn.com/abstract=2233795 (accessed May 21, 2021).

[58] Fornell C Larcker DF. Structural equation models with unobservable variables and measurement error: algebra and statistics. J Mark Res. (1981) 18:382–8. 10.1177/002224378101800313

[59] Singh R. Does my structural model represent the real phenomenon?: a review of the appropriate use of Structural Equation Modelling (SEM) model fit indices. Mark Rev. (2009) 9:199–212. 10.1362/146934709X467767

[60] Ibrahim N Shiratuddin MF Wong KW. Instruments for measuring the influence of visual persuasion: validity and reliability tests. Eur J Soc Sci Educ Res. (2015) 4:25. 10.26417/ejser.v4i1.p25-37

[61] Kim PJ Lee JY. A study on the effects of perceived quality on whitening cosmetics' satisfaction and repurchase: focused on university students. J Bus Econ Environ Stud. (2016) 6:15–22. 10.13106/eajbm.2016.vol6.no2.15

[62] Anderson EW Fornell C Lehmann DR. Customer satisfaction, market share, and profitability: findings from Sweden. J Mark. (1994) 58:53–66. 10.1177/002224299405800304

[63] Wu PCS Yeh GY-Y Hsiao C-R. The effect of store image and service quality on brand image and purchase intention for private label brands. Australas Mark J. (2011) 19:30–9. 10.1016/j.ausmj.2010.11.001

[64] Lovelock C Wright L. Principles of Service Marketing and Management. Hoboken, NJ: Prentice Hall (2001).

[65] Tsai MT Tsai CL Chang HC. The effect of customer value, customer satisfaction, and switching costs on customer loyalty: an empirical study of hypermarkets in Taiwan. Soc Behav Pers. (2010) 38:729–40. 10.2224/sbp.2010.38.6.729

[66] Lai WT Chen CF. Behavioral intentions of public transit passengers—the roles of service quality, perceived value, satisfaction and involvement. Transp Policy. (2011) 18:318–25. 10.1016/j.tranpol.2010.09.00327409075

[67] Wu LY Chen KY Chen PY Cheng SL. Perceived value, transaction cost, and repurchase-intention in online shopping: a relational exchange perspective. J Bus Res. (2014) 67:2768–76. 10.1016/j.jbusres.2012.09.007

[68] Lam LW Chan KW Fong D Lo F. Does the look matter? The impact of casino servicescape on gaming customer satisfaction, intention to revisit, and desire to stay. Int J Hosp Manag. (2011) 30:558–67. 10.1016/j.ijhm.2010.10.003

[69] Back AL Fromme EK Meier DE. Training clinicians with communication skills needed to match medical treatments to patient values. J Am Geriatr Soc. (2019) 67:S435–41. 10.1111/jgs.15709 31074864

