# Supplementary material for: Post-purchase behavioral intention in medical aesthetic: the role of image, perceived value, and satisfaction
Source: Front Public Health. 2025 Jan 22;12:1471496. doi: 10.3389/fpubh.2024.1471496 (PMC11794489; doi:10.3389/fpubh.2024.1471496)
Supplement: Supplementary file 1 [file Supplementary_file_1.docx]

Appendix

**Part I: Medical Imagery**

[Instructions] There are 8 questions in this section, which mainly aim at understanding the thoughts, attitudes and impressions brought by cosmetic medicine to you. The grading scale is "strongly agree 7, agree 6, somewhat agree 5, average 4, somewhat disagree 3, disagree 2, strongly disagree 1", the higher the score, the more you agree with the ideas, attitudes, and impressions brought by cosmetic medicine. Please fill in the answers according to your current feelings and mark "✓" in the □ column as appropriate.

| **Description**: "Aesthetic Imagery" is the thoughts, attitudes and impressions that aesthetic medicine brings to its customers. Consumers' imagery of aesthetic products and services affects their consumer decisions. |
| --- |
|  |
| 1. The decorative design of a medical aesthetic organization is visually appealing. |
| 2. The service staff of the medical aesthetic organization is attentive and friendly, showing confidence and professionalism. |
| 3. The environment and atmosphere of the medical aesthetic organization create a warm feeling, which can reduce nervousness. |
| 4. The treatments offered by medical aesthetic organizations give people more self-confidence. |
| 5. The various treatments in the medical aesthetic organization focus on and comply with the concept of patient safety. |
| 6. The professional quality of medical aesthetic organizations can be trusted. |
| 7. The services provided by medical aesthetic organizations respect the privacy and rights of patients. |
| 8. The overall image of the medical aesthetic organization is good. |

**Part II: Perceived Value**

This section consists of 6 questions, which are mainly designed to understand your overall evaluation of the effectiveness of medical products or services. The grading scale is "strongly agree 7, agree 6, somewhat agree 5, average 4, somewhat disagree 3, disagree 2, strongly disagree 1", the higher the score, the more you agree with the value added to the effectiveness of the medical aesthetic products or services, please fill in the answers one by one according to your current actual feelings, and mark "✓" in the appropriate option □.

| **Description**: "Perceived Value" refers to the consumer's overall assessment of the utility of a medical aesthetic product or service based on the perception of getting and paying for it. Consumers will use their own preferences to measure the value that has been realized in the past or will be realized in the future. |
| --- |
|  |
| 9. The services provided by the medical aesthetics organization are pleasant to you. |
| 10. The services provided by the medical aesthetic organization make you feel considerate. |
| 11. The services provided by medical aesthetic organizations are strictly regulated, so that you can have no worries.  　Heart. |
| 12. The services provided by the medical aesthetic organization can meet your needs. |
| 13. The services and quality provided by the medical aesthetic organization can meet your requirements. |
| 14. It is worth the time and money to come to a medical aesthetic organization. |

**Part III: Customer** Satisfaction

[Explanation] There are 6 questions in this section, which mainly aim at understanding your satisfaction level towards the environment, products or services provided by the medical aesthetic organizations. The rating scale is "strongly agree 7 marks, agree 6 marks, somewhat agree 5 marks, ordinary 4 marks, somewhat disagree 3 marks, disagree 2 marks, strongly disagree 1 mark", the higher the score, the more you agree with the degree of satisfaction of the products or services provided by the medical aesthetic organization, please fill in the answers according to your current actual feelings, and mark "✓" in the □ column of the appropriate option.

| **Description**: "Customer Satisfaction" refers to the psychological feeling and cognition of customers after purchasing medical and aesthetic products and services, and is a subjective and overall assessment of customers' previous experience. |
| --- |
|  |
| 15. I feel comfortable with the equipment of the medical aesthetic organization. |
| 16. I am satisfied with the ambience and comfort of the medical aesthetic organization. |
| 17. I am satisfied with the types of treatments and products offered by the medical aesthetic organization. |
| 18. I am professionally satisfied with the medical quality and technology of the medical aesthetic organization. |
| 19. I am satisfied with the professionalism of the doctors and staff of the medical aesthetic organization.  Intentions. |
| 20. I feel that the doctors and staff of the medical aesthetic organization are friendly and kind.  To Satisfaction. |

**Part IV: Post-Purchase Behavioral Intentions**

[Explanation] There are 5 questions in this section, which mainly aim at understanding your tendency to take specific actions or behaviors towards aesthetic products or companies after consumption. The grading scale is "strongly agree 7 points, agree 6 points, somewhat agree 5 points, common 4 points, somewhat disagree 3 points, disagree 2 points, strongly disagree 1 point", the higher the score, the more you agree with the degree of post-purchase behavior you may take, please fill in the answers one by one according to your current actual feelings, and mark "✓" in the □ of the appropriate option.

| **Description**: Post-Purchase Behavioral Intentions (PBI) refers to the specific actions or behavioral tendencies that customers may take towards aesthetic products or companies after they have made a purchase, which can predict whether the consumer has the potential to become a long-term customer. |
| --- |
|  |
| 21. I will tell others about the benefits of medical aesthetic organizations. |
| 22. I would recommend my friends and relatives to undergo aesthetic medicine at a medical aesthetic organization. |
| 23. Based on the results of my past aesthetic medicine practice, I am willing to come back to the medical aesthetic program.  Allow the organization to consume. |
| 24. If I have any problems with the services provided by a medical beauty care organization, I will seek advice from the service provider.  Staff Reaction. |
| 25. If there is time, I would like to receive or learn more about the medical aesthetic organization  The promotional activities organized. |

**Part V: Demographic Characterization Survey**

[Note] There are 7 questions in this section, mainly for understanding your gender, age, marital status, whether you have children, highest education level, occupation and average monthly income. Please fill in the answers one by one according to your current actual situation and tick "✓" in the appropriate option □. This information is only for data analysis purpose, and **will not be disclosed to the public**.

| 1. Gender | □ Female □ Male |
| --- | --- |
| 2. Age | □ Below 20 years old □ 21~30 years old □ 31~40 years old □ 41~50 years old □ 51~60 years old □ Above 60 years old |
| 3. Marital Status | □ Unmarried □ Married □ Other (including divorce, widowhood, separation, etc.) |
| 4. Have children | □ no children □ ___ children |
| 5. Highest academic qualification | □ Institute or above □ University □ Specialized (5 or 2)  □ High school □ Junior high school or below |
| 6. Occupation | □ Public service □ Private business service □ Free enterprise  □ Self-employed (SOHO) □ Household □ Student |
| 7. Average monthly income  (Unit: Yuan) | □ Below 20000 □ 20001~40000 □ 40001~60000  □ 60001~100000 □ Above 100000 |

**Part VI: Survey on the Characteristics of Medical Beauty Consumption**

[Note] There are 4 questions in this section, mainly to understand your consumption time, consumption place, consumption type and consumption items. Please fill in the answers one by one according to your current actual situation and tick "✓" in the appropriate option □. This information is only for data analysis purpose, and **will not be disclosed to the public**.

| 1. Last visit  Medical Time | □ Within 1 year □ 1 to 2 years (excluding 2 years) □ 2 to 3 years (excluding 3 years)  □ more than 3 years |
| --- | --- |
| 2. Content of treatment | □ Medical aesthetic organizations (including plastic surgery)  □ Medical aesthetic organizations (excluding plastic surgery) |
| 3. Consumption type | □ Project type (including treatment package) □ Single treatment type  □ Both. |
| 4. Consumption items | □ Phototherapy □ Injection □ Cosmetic Surgery (Multiple choice) |
